# Ultraviolet Irradiation-Dependent Fluorescence Enhancement of Hemoglobin Catalyzed by Reactive Oxygen Species

**DOI:** 10.1371/journal.pone.0044142

**Published:** 2012-08-30

**Authors:** Leiting Pan, Xiaoxu Wang, Shuying Yang, Xian Wu, Imshik Lee, Xinzheng Zhang, Romano A. Rupp, Jingjun Xu

**Affiliations:** 1 The Key Laboratory of Weak-Light Nonlinear Photonics, Ministry of Education, TEDA Applied Physics School and School of Physics, Nankai University, Tianjin, China; 2 Faculty of Physics, Vienna University, Vienna, Austria; Universidade Federal do Rio de Janeiro, Brazil

## Abstract

Ultraviolet (UV) light has a potent effect on biological organisms. Hemoglobin, an oxygen-transport protein, plays an irreplaceable role in sustaining life of all vertebrates. In this study we scrutinize the effects of ultraviolet irradiation (UVI) as well as visible irradiation on the fluorescence characteristics of bovine hemoglobin (BHb) *in vitro*. Data show that UVI results in fluorescence enhancement of BHb in a dose-dependant manner. Furthermore, UVI-induced fluorescence enhancement is significantly increased when BHb is pretreated with hydrogen peroxide (H_2_O_2_), a type of reactive oxygen species (ROS). Meanwhile, The water-soluble antioxidant vitamin C suppresses this UVI-induced fluorescence enhancement. In contrast, green light irradiation does not lead to fluorescence enhancement of BHb no matter whether H_2_O_2_ is acting on the BHb solution or not. Taken together, these results indicate that catalysis of ROS and UVI-dependent irradiation play two key roles in the process of UVI-induced fluorescence enhancement of BHb.

## Introduction

There would scarcely be life on earth without light. Main source of visible and ultraviolet (UV) light is the nuclear fusion reactor in the middle of our solar system. Although certain skin diseases can be healed by a proper dose of UV radiation, UV light is in general noxious [1]: For instance, it may cause sunburn, photoaging [2], skin cancers [3], or immune suppression [4]. Since increasing stratospheric ozone depletion of anthropogenic origin results in higher UV intensity to reach the ground, there is a growing concern about possible consequences and thus strong interest in research that might lead to a better understanding of the effects of enhanced UV irradiation (UVI) [5]. Several factors play an crucial role in UVI-induced different cellular physiological and pathological responses. One of them is the production of reactive oxygen species (ROS) through a photosensitized mechanism involving energy transfer by chromophores to oxygen molecules (photodynamic effect). There is a wealth of evidence that ROS trigger, among other physiological responses, also pathological ones in biological organisms [6]–[8].

As for animals and humans, UV light penetrating into the dermis of the skin can affect erythrocytes inside the capillary [9]. Recently an enhancement of autofluorescence was observed in UVI-treated erythrocytes *in vitro* and attributed to the photodecomposition of hemoglobin (Hb) [10], [11], the major component of erythrocytes. Hb contains a benzene-ring-like structure that can exhibit high photosensitive behaviors. Therefore, as an analog of photosensitizer, Hb was used as a model molecule in the study of photodynamic therapy, a prospective treatment for certain types of cancer and tumor [12]. Since Hb is vital to oxygen transport and plays an essential role in physiological and pathological processes [13], it is necessary to fully understand the interaction between UVI and Hb. To do this, we used bovine hemoglobin (BHb) as a model in the present work to investigate the relationship among UVI, formation of ROS, photodecomposition by spectroscopy *in vitro*.

## Methods

Bovine hemoglobin (BHb), hydrogen peroxide (H_2_O_2_), and vitamin C that obtained from Sigma (St. Louis, MO, USA) were dissolved in phosphate buffered saline (PBS) solution. For all experiments, the working solutions were placed in quartz cuvettes with 3 mL. UV treatment was achieved by illumination with a quartz UV sterilizing lamp (electrical power of 20 W) at a distance of 2 cm from the samples emitting essentially at 254 nm. The illumination intensity was approximately 7 mW/cm^2^ (calculated from irradiation geometry with conversion factor of 24% for UV light/electrical power). A comparison group was illuminated by an expanded beam from a 532 nm diode laser (as one representation of visible light) at 60 mW/cm^2^. Fluorescence spectra of the samples were monitored with a spectrofluorometer (FLS920, Edinburgh Instruments, U.K.) at an excitation wavelength of 365 nm. Absorption spectra were acquired with a spectrophotometer (U-4100, Hitachi, Japan).

## Results

### UVI-induced Fluorescence Enhancement of BHb

**Figure 1:**
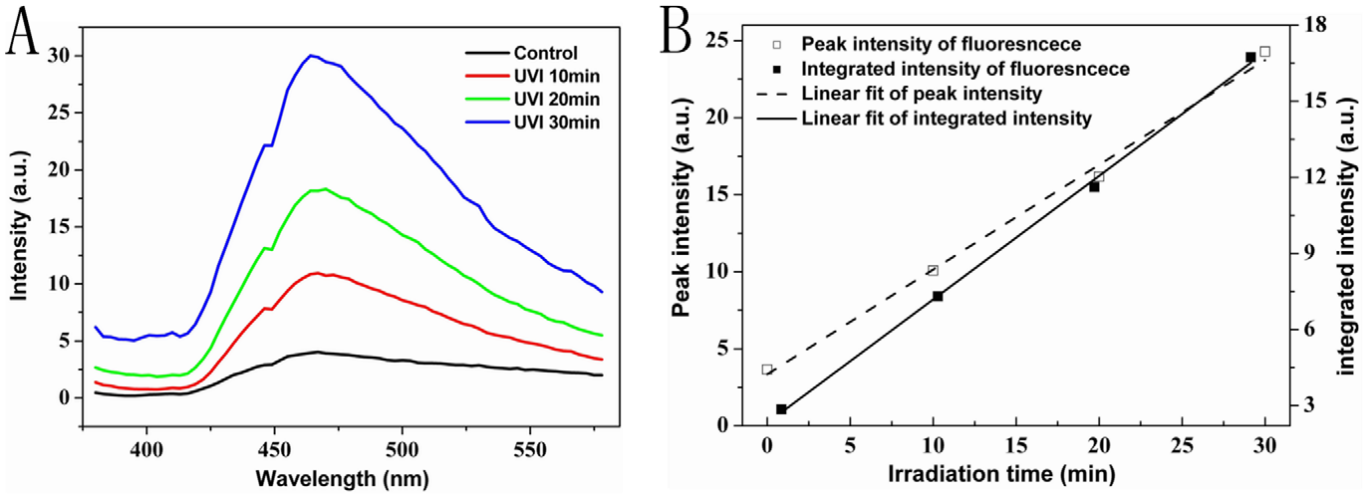
UVI induces fluorescence enhancement of BHb in a dose-dependent manner. (A) Spectra detection of a 10 µM BHb solution (at 365 nm excitation wavelength) after irradiation for 10 min (red line), 20 min (green line), and 30 min (blue line) with 7 mW/cm^2^ UV light. Black line is the fluorescence spectrum without prior UVI. (B) Intensity at the peak and integrated intensity (baseline corrected and integrated from 450 nm to 550 nm) plotted versus irradiation time.

A solution containing 10 µM BHb was irradiated with a UV sterilizing lamp for 0 min, 10 min, 20 min, and 30 min, respectively. Fluorescence emission at 365 nm excitation was recorded immediately afterwards by a spectrofluorometer (apart from the 0 min case, excitation irradiation during measurement being negligible in comparison to the prior UVI dose). For the spectra shown in Fig. 1a, the intensities of 3 scans were summed up. We could found that UVI significantly resulted in fluorescence enhancement of BHb solution in a dose-dependent manner. Besides, throughout the irradiation range investigated, we observed no saturation due to consumption of the irradiated molecules: there is a strictly linear increase of fluorescence with UVI dose (Fig. 1b). So the consumption of BHb by UVI-induced processes thus remains negligible in comparison to the total number of BHb.

### ROS-catalyzed Fluorescence Enhancement of BHb Induced by UVI

**Figure 2:**
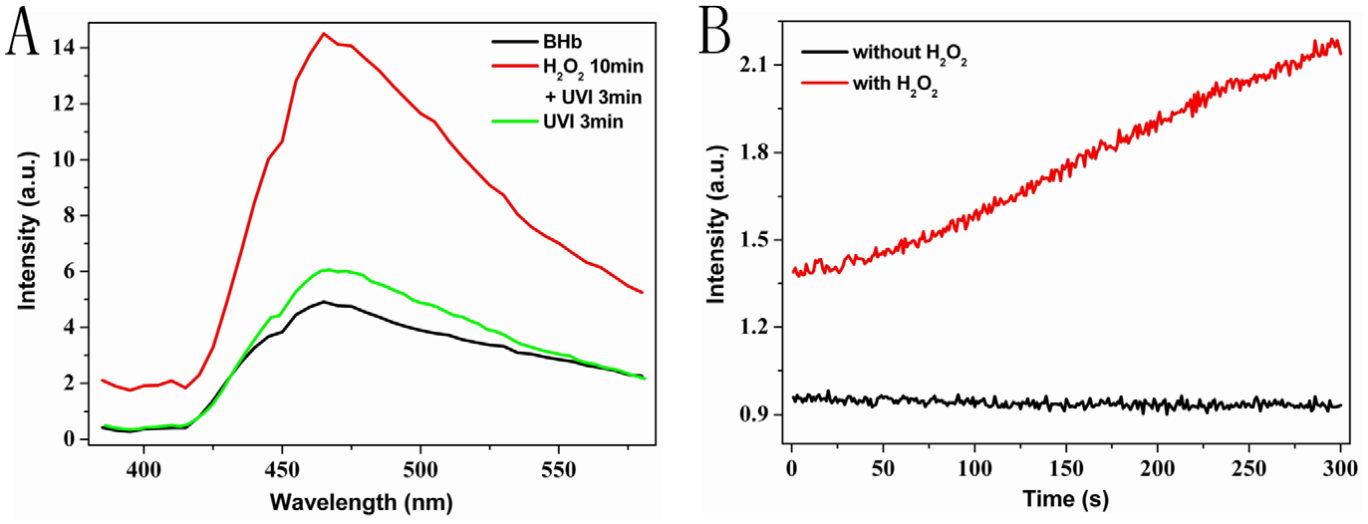
H_2_O_2_ catalyzes UVI-induced fluorescence enhancement of BHb. (A) Fluorescence spectra of different samples at 365 nm excitation: black line represents a BHb solution (10 µM) without any treatment; green line indicates the UVI-pretreated (3 min) BHb solution; red line corresponds to H_2_O_2_-preincubated (1 mM for 10 min) BHb solution with subsequent UVI treatment (7 mW/cm^2^, 3 min). (B) Real-time monitoring of fluorescence at 464/2 nm for a sample with or without 1 mM H_2_O_2_ treatment at 365 nm excitation. Black line corresponds to the pure BHb solution, red line indicates BHb solution pretreated with 1 mM H_2_O_2_ for 10 min.

It is well known that ROS plays a crucial role in various UVI-induced different biological effects [6]–[8]. Therefore, we needed to figure out whether ROS was involved in this process of UVI-induced fluorescence enhancement. First, 1 mM H_2_O_2_, one kind of ROS, was selected to preincubated the BHb solution (10 µM) for 10 minutes in the dark, Then, this H_2_O_2_-pretreated BHb solution was immediately irradiated by UV light for another 3 min. It was clearly that BHb solution pretreated with H_2_O_2_ showed much higher fluorescence intensity at the same UVI irradiation time (Fig. 2a), implying that ROS could catalyze UVI-induced fluorescence enhancement of BHb. To further determine the catalytic effect of ROS, low-dose UV excitation light (365 nm) from spectrofluorometer was used to irradiate the BHb solution with or without pretreatment with H_2_O_2_. Under this condition, the effect of ROS made by UV excitation itself can be neglected. Therefore, this UV excitation light had two roles in the process of fluorescence change: to serve as excitation light for fluorescence detection and to provide low-dose UVI for treating samples. We monitored the dynamic variation of the samples fluorescence intensity with spectrofluorometer in a real-time manner with 300 s. As summarized in Fig. 2b, this low-dose UV excitation light resulted in a linear increase of fluorescence of H_2_O_2_-pretreated BHb solution, whereas it did not have this increase effect without the assistance of H_2_O_2_. These data suggested that ROS revealed a catalytic role in this process of fluorescence enhancement.

### The Protection Role of Vitamin C for UVI-induced Phototoxic Effect

**Figure 3:**
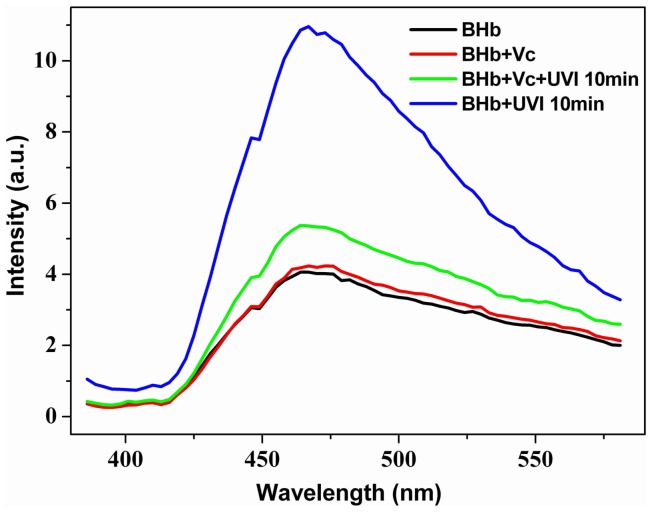
Vitamin C suppresses UVI-induced fluorescence enhancement of BHb. Black line indicates a pure BHb solution (10 µM); red line represents the mixture of BHb (10 µM) and vitamin C (10 mM); green line corresponds to the mixture of BHb (10 µM) and vitamin C (10 mM) after UVI for 10 min; blue line indicates UVI-pretreated (10 min) BHb solution.

To further confirm whether ROS was necessary, the potent antioxidant vitamin C was used in this work. Vitamin C (10 mM) was added to BHb (10 µM) solution for study. As shown in Fig. 3, the fluorescence spectra of pure BHb solution and BHb solution with vitamin C are identical (black line and red line). Then, we irradiated this mixture solution for 10 min using UV sterilizing lamp. Data showed that, after treatment with UVI for the same time, the fluorescence intensity of BHb solution mixed with vitamin C (green line) was markedly lower than that of pure BHb solution (blue line), indicating the obvious protection role of vitamin C.

### UVI-dependent Fluorescence Enhancement of BHb

**Figure 4:**
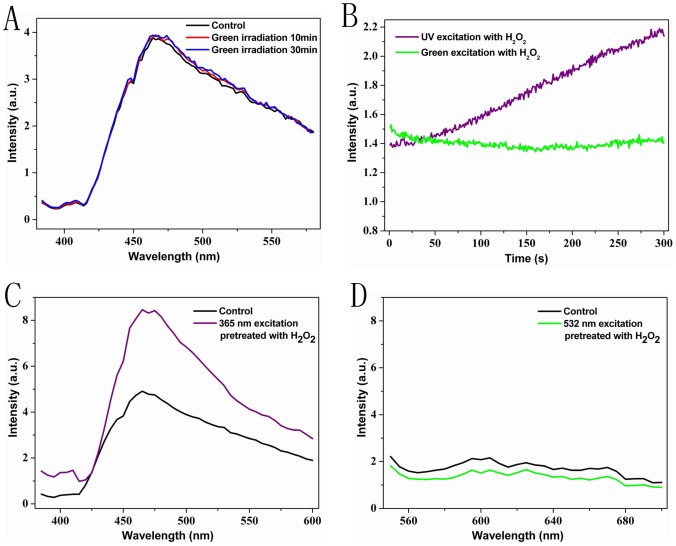
Fluorescence enhancement of BHb depends on UVI. (A) Fluorescence spectra of 10 µM BHb solution (365 nm excitation) after irradiation with 532 nm laser at an intensity of 60 mW/cm^2^ for 0 min (black line), 10 min (red line), and 30 min (blue line), respectively. (B) Real-time detection of fluorescence of 1 mM H_2_O_2_-pretreated (10 min) BHb solution at 595/2 nm with 532 nm excitation (green line) and at 464/2 nm with 365 nm excitation (purple line). (C) Fluorescence spectra of BHb solution with 365 nm excitation: black line means the pure BHb solution, purple line indicates BHb solution pretreated with 1 mM H_2_O_2_ for 10 min. (D) Fluorescence spectra of BHb solution with 532 nm excitation: black line corresponds to a BHb solution without any treatment, green line represents the BHb solution pretreated with 1 mM H_2_O_2_ for 10 min.

It was reported that visible light could also result in ROS generation and stimulate biological effects in various cell types [14], [15]. For that reason we were also searching for fluorescence responses to irradiation by visible light. A 60 mW/cm^2^ green laser (532 nm) was selected to be a representative visible light source for the irradiation of 10 µM BHb solution. As shown in Fig. 4a, no fluorescence enhancement is found by green irradiation of BHb solution. Monitoring real-time fluorescence intensity of H_2_O_2_-pretreated BHb solution at 595/2 nm for low-dose excitation at 532 nm did not have any effect on fluorescence enhancement (green line in Fig. 4b) despite of a pretreatment with H_2_O_2_ while dim UV excitation stimulates increase of fluorescence under the same conditions (purple line in Fig. 4b). Likewise, there is fluorescence enhancement upon low-dose UV excitation light that is increased by action of ROS, as already discussed, however no such effect can be found for low-dose green excitation light (Fig. 4c,d). These results clearly demonstrated that fluorescence enhancement of BHb solution depended on UVI.

### UV/Vis Absorption Measurement of Samples

**Figure 5:**
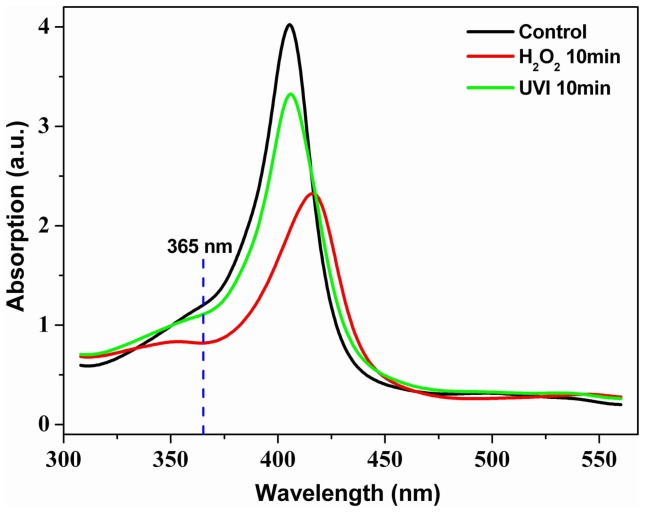
UV/Vis absorption spectra of the samples. Black line indicates the pure BHb solution (10 µM); green line represents UVI-pretreated (10 min) BHb solution; red line corresponds to BHb solution pretreated with 1 mM H_2_O_2_-pretreated for 10 min. Dashed line indicates the position of the excitation wavelength of the fluorescence measurements.

Further studies were needed to confirm whether the UVI-induced fluorescence enhancement of BHb was due to the higher fluorescence efficiency of photodecomposition products of BHb because increasing absorption of excitation light maybe also result in strong fluorescence. Therefore, we measured the UV/Vis absorption spectra of different samples. Figure 5 showed the UV/Vis absorption spectra of pure BHb solution (black line), UVI-pretreated BHb solution (green line), and H_2_O_2_-pretreated BHb solution (red line), respectively. It was obvious that the effect of UVI or H_2_O_2_ resulted in a decrease of absorption rate around 365 nm, supporting the production of BHb photodecomposition products with higher fluorescence efficiency.

## Discussion

Data clearly showed that UVI could result in fluorescence enhancement of BHb solution in a dose-dependent manner by spectroscopy method (Fig. 1). Kaestner et al. [10] observed an enhancement of autofluorescence in UVA-treated (380 nm) erythrocytes and attributed the increase of fluorescence to fluorescent bilirubin generated by UV-induced photodecomposition of Hb with the argument that the form as well as the wavelength of the maximum of the fluorescence spectrum agrees reasonably with the one reported for isolated bilirubin [16]. It was found that apart from a small indentation at about 450 nm the form of the spectra with a steeper slope on the shorter wavelength side than on the long wavelength side of the maximum is similar to the one shown in [10] for human erythrocytes, however the maximum is at a significantly shorter wavelength, namely 470 nm instead of 508 nm, indicating that fluorescent molecules generated by UV-induced photodecomposition of BHb solution may be different from that of erythrocytes.

UVI can generate large amounts of ROS through photodynamic effect. Meanwhile, it is well known that ROS plays an important role in UVI-induced many biological effects [6]–[8]. The question remains, whether the fluorescent molecules are generated directly or whether they result indirectly from photo-generated ROS. In the latter case photo-induced fluorescence is a secondary consequence of primary ROS photo-production. This second pathway is the so-called photodynamic effect. In particular, ROS induces structural changes of hemoglobin [17], [18] and participates in the degradation process [19], [20]. In a further series of experiments we therefore targeted to test for involvement of ROS in the observed UVI-induced fluorescence enhancement. To that aim, we have chosen H_2_O_2_, a frequently occurring ROS. As illuminated in Fig. 2a, H_2_O_2_-preincubated (1 mM for 10 min) BHb solution with subsequent UVI treatment (red line) show much higher fluorescence than that of only UVI-preincubated sample (green line). We propose to explain this result by the hypothesis that UVI-induced enhancement of fluorescence is catalyzed by ROS. To further investigate the catalytic effect of ROS, we consider the effect of UV excitation at 365 nm. Besides its role as excitation source during the fluorescence measurement run, it acts in the same time as a low-dose irradiation source. This is to be expected, since ROS production by this low-dose irradiation source at 365 nm is negligible. In a real-time fluorescence measurement we followed the fluorescence without and with H_2_O_2_ pretreatment at 465 nm as a function of time over 300 s. As can be seen from Fig. 2b for the sample without H_2_O_2_, there is no increase of fluorescence. However, one can see a steady increase of fluorescence for the ROS-pretreated solution. In this result we see a confirmation of our view that ROS plays a catalytic role in this process of UV-induced fluorescence enhancement.

Nagababu et al [20] identified two fluorescence bands to appear upon chemical attack by ROS in Hb solution, one having emission centered at 465 nm with 321 nm excitation and one having emission centered at 525 nm with excitation maximum at 460 nm. From the differing time course of the bands it was concluded that they belong to two different compounds. Comparing the ROS-induced fluorescence bands with our UVI-induced bands, one can see that the ROS-induced band with emission at 525 nm can be identified with the UVI-induced fluorescence band in human erythrocytes observed by Kaestner et al. and thus can be attributed to bilirubin, while the band with emission at 465 nm is identical with the UVI-induced band in BHb solution observed in our study and belongs to a hitherto unidentified compound. The most plausible conclusion that can be drawn from these facts is that the same fluorescent molecules are generated by ROS and by UVI. This suggests that the UVI-generated fluorescent species is due to a photodynamic mechanism involving UV generation of ROS in the first step followed by a secondary generation of the fluorescent species by the action of ROS.

Vitamin C reduces UVI-induced damage in all animals and protects skin from photoaging and cancer [21], [22] because it is a potent antioxidant [23]. Specially, vitamin C contribute to the decrease in the oxidative stress of erythrocytes [24]. In order to further submit the hypothesis of ROS involvement in the effect of UVI, we added vitamin C (10 mM) to BHb solution. The fluorescence spectra of pure BHb solution and BHb solution with vitamin C are identical (black line and red line in Fig. 3). If we compare now the effect of UVI by the UV sterilizing lamp for 10 min on theses two samples, we see that the effect of fluorescence enhancement is significantly suppressed by vitamin C (Fig. 3, green line in comparison with the blue line). We see in these findings a confirmation of our assumption that ROS plays a catalytic effect in the process of UVI-induced fluorescence enhancement. In addition, we have used other ROS scavengers, such as superoxide dismutase, glutathione and catalase. It was found that these scavengers did not block fluorescence enhancement of BHb induced by UVI *in vitro* (data not shown). It seems that vitamin C play a special role in protection against UVI-induced fluorescence enhancement of Hb. Further study is still needed to elucidate the reason for different effects of these ROS scavengers on UVI-induced fluorescence enhancement of Hb.

Furthermore, some studies reported on ROS generation by visible light and its stimulation of biological effects in various cell types [14], [15]. For that reason we were also searching for fluorescence responses to irradiation by visible light. A 60 mW/cm^2^ green laser (532 nm) was selected to be a representative visible light source for the irradiation of 10 µM BHb solution. As shown in Fig. 4a, no fluorescence enhancement is found by green irradiation of BHb solution. Monitoring real-time fluorescence intensity of H_2_O_2_-pretreated BHb solution at 595/2 nm for low-dose excitation at 532 nm did not have any effect on fluorescence enhancement (green line in Fig. 4b) despite of a pretreatment with H_2_O_2_ while dim UV light stimulates increase of fluorescence under the same conditions (purple line in Fig. 4b). Likewise, there is fluorescence enhancement upon low-dose UV excitation light that is increased by action of ROS, as already discussed, however no such effect can be found for low-dose green excitation light (Fig. 4c,d). Taken together, These results clearly demonstrated that fluorescence enhancement of BHb solution depended on UVI.

Besides, Figure 5 compares the UV/VIS absorption spectra of native BHb solution (black line) with UVI-pretreated BHb solution (green line) and H_2_O_2_-pretreated BHb solution (red line), respectively. There are two similarities: Both, UVI as well as chemical treatment with H_2_O_2_ lead to a decrease of two absorption bands, one around 410 nm and one around 360 nm (only seen as a shoulder of the former band). However, there is also a remarkable difference: while a new band appears at about 425 nm for ROS treatment, this does not happen for UV treatment. We may then roughly sketch the situation at the excitation wavelength 365 nm as follows: The absorption at 365 nm can be thought to consist of two contributions, one proportional to the number density *N_1_* of molecules with high fluorescence efficiency for 460 nm emission and one proportional to the number density *N_2_* of non-fluorescent molecules (or molecules of very low fluorescence efficiency). Above we established that fluorescence at 365 nm excitation is increased by UVI as well as by chemical ROS attack. If *N_2_* were constant, one would therefore expect an increase in absorption at 365 nm – which is not the case. It is therefore beyond doubt that *N_2_* decreases due to UVI or chemical ROS attack, subsequently resulting in the generation of *N_1_*. therefore, when *N_1_* increases, the fluorescence intensity simply increases because more photons are available for fluorescence-effective absorption due to elimination of concurrent non-fluorescent processes.

In conclusion, data show that UVI induces fluorescence enhancement of BHb solution with increasing irradiation dose while irradiation with visible light does not. We suggested that the higher single photon energy of UV light results in photodecomposition of Hb, which is not the case for photons of green light. Furthermore, ROS generated by UVI plays an obviously catalytic role in the process of fluorescence enhancement. Fluorescence enhancement of BHb is due to the catalysis of ROS and UVI-dependent photodecomposition. As a physical therapy, ultraviolet blood irradiation and oxygenation (UBIO) was used to treat many nonspecific diseases in clinics [25], [26]. Especially, UBIO could improve the oxygen transport function of erythrocytes [25], [27]. We can see that Hb may play an important role in this therapy because it is the main target in UVI-treated erythrocytes. Therefore, We believe that this study of UVI-induced enhancement of BHb might fill an important gap in the research of UBIO.
